# Cognitive function assessment during 2 mA transcranial direct current stimulation in DLPFC in healthy volunteers

**DOI:** 10.14814/phy2.14264

**Published:** 2019-10-29

**Authors:** Shahid Bashir, Fawaz Al‐Hussain, Ali Hamza, Talha Asim Niaz, Raidah Albaradie, Syed S. Habib

**Affiliations:** ^1^ Neuroscience Center King Fahad Specialist Hospital Dammam Dammam Saudi Arabia; ^2^ Department of Neurology College of Medicine King Saud University Riyadh Saudi Arabia; ^3^ Department of Electrical Engineering National University of Computer and Emerging Sciences Lahore Pakistan; ^4^ Department of Physiology College of Medicine King Saud University Riyadh Saudi Arabia

**Keywords:** Memory, safety, stop signal task, transcranial direct current stimulation

## Abstract

Although cognitive function has been reported to change following the anodal transcranial direct current stimulation (tDCS) but still variable results have been reported in healthy subject and there is paucity of data on the cognitive effects of online tDCS. Therefore, we aimed to assess the online effect of tDCS over the left dorsolateral prefrontal cortex (DLPFC) on cognitive function and obtain safety data in healthy adults. We recruited 36 healthy (20 male) participants for this double‐blind, sham‐controlled parallel design. We used Stop Signal Task (SST) Go Trial and Pattern Recognition Memory (PRM) tests to evaluate cognitive function during 2 mA (20 min) anodal or sham tDCS stimulation over the left DLPFC. In active conditions, left dorsolateral prefrontal cortex was selected for electrode placement with reference over right supraorbital cortex. All related tasks were done during the online tDCS section in both groups (active/sham). There were statistically significant differences in cognitive function according to the PRM test (*P* = 0.003), SST (*P* = 0.021), and SST correct response time on Go Trials (*P* = 0.02) during active stimulation compared to the sham group. Our results reveal that cognitive performance is affected by a single dose of active online tDCS over DLPFC area compared to sham stimulation. In our study, tDCS is well‐tolerated and safe that further supports the safety of tDCS in local healthy population.

## Introduction

Transcranial direct current stimulation (tDCS) is a non‐invasive and painless neurostimulation technique that modulates brain activity; more specifically, anodal stimulation increases cortical excitability and cathodal stimulation decreases it (Nitsche and Paulus 2000; Nitsche et al. 2003, 2004).

There is developing evidence that tDCS of the dorsolateral prefrontal cortex (DLPFC) improves cognitive performance both healthy individuals and patients suffering from various neurological diseases (e.g., on working memory training; (Coffman and Parasuraman 2014; Brunoni and Vanderhasselt 2014; Horvath et al. 2014). Such positive tDCS effects in single and multiple session on cognitive performance over several days (Reis et al. 2008; Thomson 2010; Boggio et al. 2010; Bolognini et al. 2010; Coffman et al. 2012), with an improvement of up to 3 months Reis et al. (2008). It has been reported in a recently published meta‐analysis that results related to behavior after single tDCS sessions produce different results and may not have any effect on cognition. (Horvath et al. 2015; Cinel et al. 2019). Therefore, the potential of tDCS to modulate brain activity and cognitive function remains a matter of controversy and questionable. It is highly likely that tDCS effects are state‐dependent with aa time scale ranging from minutes to hours, and is highly dependent on the immediate history of neural circuits activity (Silvanto et al. 2008) and occurrence of synaptic plasticity (Karabanov et al. 2015; Ziemann and Siebner 2015).

With the advent of modern computerized cognitive testing batteries such as Cambridge neuropsychological test automated battery (CANTAB) it has been possible with certainty to allow for hypothesis‐driven exploration of different domains of cognition testing (Bashir et al. 2017; Al Backer et al. 2018; Habib et al. 2018; Al‐Thaqib et al. 2018; Alghamdi et al. 2019). CANTAB batteries include a wide variety of tests, for example working memory testing with pattern recognition Memory (PRM) and the Stop Signal Test (SST), for executive functions and measure decision‐making ability, and response inhibition. We hypothesize that while the anodal stimulation over left DLPFC may improve subsequent inhibition performance assessed by SST.

There is no sign for irreversible brain damage produced by tDCS protocols within a wide range of stimulation parameters (≤40 min, ≤4 mA, ≤7.2°C) (Bikson et al. 2016). The purpose of the study was to evaluate the influence of online anodal 2 mA tDCS over the left DLPFC area and its effect on cognition using the SST PRM tasks from the CANTAB. Second, to study the safety and tolerability aspects of 2 mA tDCS by adverse events questionnaire in healthy adults compared with sham stimulation.

## Methods

### Subjects

This study was conducted on 36 (20 males) subjects who were recruited, which was a randomized, parallel experimental double‐blind, sham‐controlled in which subjects received one of two randomly assigned tDCS conditions: anodal over left DLPFC (cathodal over right supraorbital), or sham. At each visit, participants received two 20‐min stimulation sessions with cognitive testing performance. The mean age of the participants was 24.3 ± 5.03 years (Table 1). All procedures were conducted according to the Declaration of Helsinki.

**Table 1:** Participant demography and clinical characteristics

Group	Active	Sham	*P* value
Age	22.87 ± 3.8	22.40 ± 2.0	0.98
Education years	13.00 ± 2.2	13.22 ± 2.4	0.82
MMSE	28 ± 1.2	28 ± 1.1	0.96

Data are expressed as Mean ± SD. Data were compared using Student’s *t* tests.

SD, standard deviation; MMSE, mini mental state exam

Participants were signed on informed written consent and screened before the first session from contraindications (Bikson et al. 2016). No subject had ever received any previous brain modulation. Informed written informed consent was obtained from all subjects, who received explanation of the purpose of study and potential side effects, before participation. All subjects have given their informed consent and that the study protocol has been approved by the institute's committee on human research at King Khalid University Hospital.

We screened all the subjects before performing neurocognitive assessments with a validated Mini‐mental State Examination (MMSE) in Arabic language (Vertesi et al. 2001, Ibn Yacoub et al. 2012). The main components of MMSE are orientation section with a maximum of 10 points, memory part with a maximum of 3 points, attention and calculation part with 5 points, 9 points for language competence and 3 points for recall. The score of maximum is 30 with <27 points is considered cognitive impairment.

### Inclusion and exclusion criteria

Subjects were required to be ≥18 years of age, with no history of neurological or psychological disorders, such as epilepsy or stroke, or any previous head surgeries. Participants who presented with any skin disorder at or near stimulation locations (i.e., where the electrodes were to be placed), such as eczema, rashes, or other skin defects ramp up and ramp down over 15 sec, were excluded.

### tDCS

All subjects were first seated comfortably. We used StarStim NE noninvasive wireless t‐DCS neurostimulator (NE Neuroelectrics®, Barcelona, Spain)for DC current delivery. It encompassesa wireless neoprene cap, applied on scalp according to 10–20 international system. Small Ag/AgCl gelled electrodes, with a surface contact area of 3.14 cm^2^ specific to the StarStimNE device (Pi electrodes, Neuroelectrics^®^), were placed over the left DLPFC at F3 (anodal) and area, (C4; return electrode). The electrodes were linked to a control box device, which was wirelessly connected to a with the NIC software (version 1.2, Neuroelectrics^®^). During anodal stimulation at 2‐mA intensity and applied for SST and PRM task duration within the control box device that delivered from a current‐control circuit in the battery‐driven stimulator. For the sham stimulation, electrodes were placed in the similar position and participants received a short ramp up (20s total up/down) at the beginning and end of the stimulation period.

### Cognitive function

Neuropsychological testing was performed two times during tDCS stimulation (online) using CANTAB research suite software (version 6. 0.37, Cambridge Cognition, Cambridge, United Kingdom). The selected tests in the battery required 15 to 20 min to complete the tasks. Subject was asked to sit comfortably on a stool and keep pressing the response button with his/her index finger of the dominant hand, according to manufacturer’s instructions.

### Stop signal test (SST)

The SST measured response inhibition (impulse control) by respond to an arrow stimulus of two choices depending on the direction in which the arrow points through touch screen system. The subject must inhibit that response during an audio tone in task. Therefore, this test contained of two parts. In the first part, the subject was presented with stimulus of left‐pointing arrow on screen and told to press the left‐button and when they see stimulus of right‐pointing arrow then the right‐button on the press pad. There was one block of 16 trials for the participant to practice this task. In the second part, the participant was told to continue pressing the buttons on the press pad when they see the arrows, as before; however, if they heard an auditory signal (a beep), they must withhold their response and not press the button. Which this test cover direction error and looks for the rate of successful stops, response time on Go trials and stop signal reaction time.

### Pattern recognition memory (PRM)

The PRM is a two‐choice, forced discrimination paradigm to test of visual pattern recognition memory. In the task, a sequence of visual patterns, which cannot easily be given verbal labels, was presented in the center of a screen. In the recognition phase, the subjects are required to choose between a pattern they have already seen and a novel pattern.

### tDCS adverse effects questionnaire

At each session, subjects completed the standardized questionnaire to evaluate potential adverse effects of tDCS (a headache, neck pain, mood alterations, and seizures) on a 5‐point scale. The scale also was administered at the follow‐up of tDCS stimulation.

### Data analysis

The SST has five outcome measures covered direction errors, the proportion of successful stops, RT on GO trials, SSD (50%), and SSRT. Data were analyzed using SPSS version 21.0 (IBM Corporation, Armonk, NY) for Windows (Microsoft Corporation, Redmond, WA, USA). Categorical data were expressed as absolute numbers and percentages. Numerical data were expressed as mean and standard deviation (SD); a two‐tailed *P* < 0.05 was considered to be statistically significant. To measure the acute effects of stimulation on cognitive function, ANOVA for condition (sham/active)] were performed on all primary outcome measures between‐subject factors. Follow‐up *t* tests were then used to investigate the effects of the within‐ and between‐subject factors.

## Results

### Demographic characteristics

There were 18 (mean age, 22.87 ± 3.8 years) subjects in the active group and 18 (mean age, 22.4 ± 2.6 years) in the sham group. There was no statistical difference in age between the two groups (i.e., *P* > 0.05, Table 1). The subjects were in two groups matched for MMSE scores (*P* = 0.96).

### CANTAB

**Figure 1:**
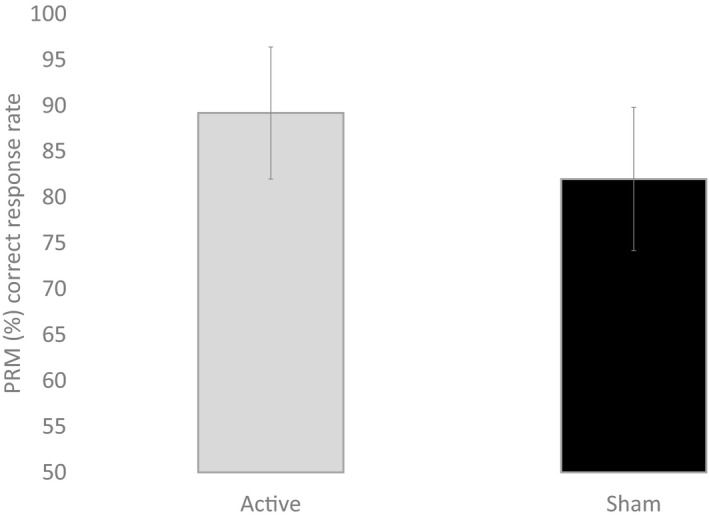
Response time (msec) from stop signal task (SST) for Go trials and stop signal correct reaction time on go trial during active anodal and sham stimulation. Error bars are standard deviation

**Figure 2:**
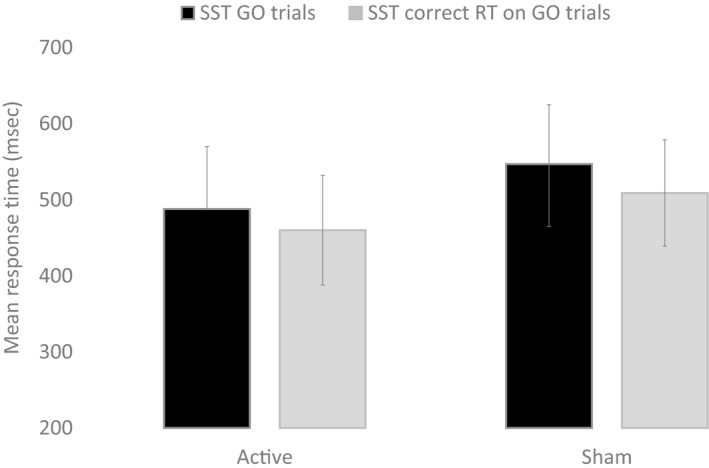
Correct response (%) from pattern recognition memory task during active anodal and sham stimulation. Error bars are standard deviation

The first objective was to test whether the cognitive performance was performed better during active stimulation compared to the participants that received sham stimulation (Table 2). A main effect of condition (active vs. sham) was observed for PRM (*P* = 0.003, Fig. 1), SST Go (*P* = 0.021) and SST correct response time (in msec) on Go Trials (*P* = 0.022, Fig. 2, Table 2).

**Table 2:** Comparison between in active and sham groups in the mean difference of cognitive function

	Anodal	Sham	*P*
	Mean ± SD	Mean ± SD	
PRM Percent correct	89.2 ± 7.2	82.0 ± 7.8	0.003
SST SSRT (last half)	172.0 ± 44.2	1.96.8 ± 46.3	0.154
SST GO trials	488.2 ± 82.6	547.1 ± 78.6	0.021
SST correct RT on GO trials	460.3 ± 72.4	509.6 ± 70.4	0.022
SST direction	1.77 ± 0.98	1.86 ± 0.10	0.307
SST proportion	0.52 ± 0.12	0.6 ± 0.8	0.191
SST SSD	261.2 ± 46.2	289.2 ± 60.2	0.149

Data were compared using Student’s *t* tests.

PRM, pattern recognition memory (%); SST, Signal Stop Task; SSRT, stop signal reaction time; SSD, stop signal delay; SST, Stop Signal Task; SD, standard deviation.

### Safety

In present experimental paradigm for stimulation was well tolerated by the participants, and no sessions were stopped due to adverse effects. Table 3 showed the occurrence of adverse effects with the mean difference in intensity for the active and sham groups. The active group, showed a significant difference (*P* value = 0.002) for headache, (*P* value = 0.043) for itching, and (*P* value = 0.025) for laziness and somnolence after giving active stimulation only. While in the sham group there were no significant differences (Table 3).

**Table 3:** Comparison between pre‐ and poststimulation in active and sham groups in the mean difference of safety

	Pre and post (mean)	*P*‐value
Active group (*n* = 18)		
Numbness	−0.067	0.328
Itching	−0.25	0.043
Pain in the neck	−0.057	0.426
Laziness and somnolence	−0.42	0.025
Headache	−0.5	0.002
Sudden changes in mood	−0.13	0.266
Change in strength of vision	−0.13	0.377
Annoying sensation	0.09	0.575
Strange sense of vision	−0.17	0.103
Sham group (*n* = 18)		
Numbness	−0.42	0.344
Itching	−0.48	0.178
Pain in the neck	0.21	0.502
Laziness and somnolence	−0.32	0.168
Headache	−0.21	0.360
Sudden change in mood	−0.26	0.254
Change in strength of vision	−0.34	0.466
Annoying sensation	0.18	0.584
Strange sense of vision	−0.26	0.286

## Discussion

In this study, healthy control performed significantly better during active anodal stimulation for SST and PRM of compared with sham stimulation. These findings are in line with previous tDCS studies involving healthy subjects have demonstrated positive changes in attention and memory (Reis et al. 2008; Sparing et al. 2009; Bolognini et al. 2010).

The cognitive performance improved in active condition only compared to sham (placebo) stimulation. The placebo effect might not be present due to the active role of brain during stimulation. Future studies should use online (task‐concurrent) tDCS approach. The main advantage of this approach is that in this montage, all participants were performing same tasks at the same time when they are receiving active tDCS to control the brain state dependence. Recent researches suggest that cognitive functions may be significantly enhanced with this paradigm (Au et al. 2016; Ruf et al. 2017; Oldrati et al. 2018). Au et al. (2016), for example, showed an active tDCS enhanced cognitive performance (7 days of working memory training) compared to sham tDCS to a sample of healthy individuals and lasted for many months after cessation of training (Au et al. 2016). In a similar research study by Ruf et al. (2017 showed improvement for spatial and verbal working memory after active tDCS in healthy adults for three consecutive training sessions.The idea of the performing task during stimulation to control the variability and the expected effects of this anodal stimulation might have increased cognitive performance in the present study.

Our study conveys an important message that it is essential to control for brain state effect and double blind design in brain stimulation studies^ (^Silvanto et al. 2008; Karabanov et al. 2015; Ziemann and Siebner 2015; Zhang et al. 2019). Brain state dependency effects may lead to obstacles for transcrinal electrical stimulation (tES) studies, particularly those with no clearly identified mechanisms like tDCS (Silvanto et al. 2008; Karabanov et al. 2015; Zhang et al. 2019). The betterment of cognition after tDCS interventions has not yet been critically addressed for its identified mechanism of action in brain. In this study, we show a clear evidence of engage brain during stimulation effects leading to improvement in cognitive function for SST task in active condition only compared to placebo effects in healthy subjects.

Safety and toxicity are additional important major concerns with regard to online tDCS that must be addressed for healthy subjects. Although tDCS differs in many aspects from other non‐invasive tES therapies for weak electric currents which does not induce directly neuronal action potentials. It has been used worldwide in thousands of subjects, with no reports of any toxic effects til date (Hu et al. 2018; Kumar et al. 2019; Bello et al. 2019). Therefore, addressing tDCS dosage parameters which are: current dosage (measured in amperes); duration of stimulation; and electrode montage (size and position of all electrodes) is critical for a safe application of tDCS.

The present study does not explore the whole understanding of this field; instead, it allows us to give a message for existing limitations of current research, which require further explorations. Limitation of the study include lack of variation in age and a large sample to detect a large significant effects of tDCS in healthy subjects.

## Conclusion

tDCS is a putative candidate to improve cognitive functions, such as memory, despite some side effects that need to be studied further. A limitation of this study was the limited time to control all the variables. A future direction of tDCS studies in our population was to investigate the physiology of the brain during a stimulation session by application of electroencephalography. However, societal acceptance of such types of treatment for neuropsychiatric disorders needs to be addressed.

## Conflicts of Interest

The authors declare no conflicts of interest.
